# Factors associated with predicting knee pain using knee X-ray and personal factors: A multivariate logistic regression and XGBoost model analysis from the Nationwide Korean Database (KNHANES)

**DOI:** 10.1371/journal.pone.0314789

**Published:** 2024-12-02

**Authors:** Taewook Kim

**Affiliations:** Department of Orthopedic Surgery, Seoul National University College of Medicine, Seoul, South Korea; Stanford University, UNITED STATES OF AMERICA

## Abstract

With increasing life expectancy, knee pain has become more prevalent, highlighting the need for early prediction. Although X-rays are commonly used for diagnosis, knee pain and X-ray findings do not always match. This study aims to identify factors contributing to knee pain in individuals with both normal and abnormal knee X-ray results to bridge the gap between X-ray findings and knee pain. Data from the fifth Korea National Health and Nutrition Examination Survey (KNHANES), collected from 2010 to 2012, including data from 5,191 participants, were analyzed. The focus was on epidemiological characteristics, medical histories, knee pain, and X-ray grades. Multivariate logistic regression and extreme gradient boosting (XGBoost) models were used to predict knee pain in individuals with normal and abnormal knee X-rays, categorized by Kellgren-Lawrence grades. For normal X-rays, the logistic regression model identified aging, being female, higher BMI, lower fat percentage, osteoporosis, depression, and rural living as factors associated with knee pain. The XGBoost model highlighted BMI, age, and sex as key predictors, with a feature importance >0.1. For abnormal X-rays, logistic regression indicated that aging, being female, higher BMI, osteoporosis, depression, and rural living were associated with knee pain. The XGBoost model highlighted age, BMI, sex, and osteoporosis as key predictors, with a feature importance >0.1. Aging and being female were associated with knee pain due to hormonal changes in women, as well as cartilage and bone deterioration. Lower fat percentage was significantly associated with increased pain, which might be attributable to higher activity levels. Higher BMI and osteoporosis were significantly associated with knee pain, possibly due to increased stress and reduced resistance on knee structures, respectively. Depression was identified as a key predictor of knee pain in patients with normal X-rays, potentially attributable to psychosomatic factors. The study’s limitations include its cross-sectional nature, which does not allow for the establishment of causal relationships, the lack of detailed medical history such as trauma history, and recall bias due to self-reported questionnaires. Future research should address these limitations to support our hypothesis.

## Introduction

Knee pain is a multifaceted symptom characterized by various anatomical and physiological changes in the knee joint, including bony abnormalities and soft tissue injuries [1, 2]. Symptoms associated with knee pain, such as stiffness, swelling, and limitations in joint function significantly impact daily activities and overall quality of life [3].

With the increasing proportion of the aging population, knee pain has become one of the most prevalent medical conditions among older adults [4, 5]. Worldwide estimates suggest that 9.6% of men and 18.0% of women develop knee pain by the age of 60 years. In the United States, the prevalence of knee pain adjusted for age and body mass index increased by approximately 65% between 1974 and 1994. The United States spends an estimated amount of $139.8 billion annually on outpatient knee pain care [6]. Given the substantial burden on health insurance and medical costs, early prediction and prevention of knee pain have become crucial [7].

In clinical practice, plain film radiography remains the primary method for evaluating knee pain-related diseases. While advanced imaging techniques such as computed tomography (CT) and magnetic resonance imaging (MRI) offer more detailed views, conventional radiography is valued for its cost-effectiveness and ability to reveal abnormal changes in the knee [8, 9]. However, not all conditions that cause knee pain are detectable on X-rays. For instance, osteoarthritis typically shows visible signs like joint space narrowing and osteophyte formation [10], whereas meniscus injuries might not be visible on X-rays, despite causing significant pain and discomfort [11]. Consequently, relying solely on conventional radiographs to diagnose knee pain can be challenging [12].

This study aims to predict situations where knee pain may occur. We hypothesize that multiple factors influence the relationship between X-rays and knee pain. To explore these factors, we utilize both a classical multivariate logistic regression model and a modern machine learning technique known as Extreme Gradient Boosting (XGBoost). XGBoost is a machine learning method that combines the predictions of multiple small decision trees to enhance accuracy. By aggregating the results of multiple trees, the model gradually improves its accuracy. This method works by iteratively combining the outputs of several weak learners (individual decision trees) to create a robust, accurate predictive model [13].

Given that most clinical settings only have access to X-rays, this research is significant because it provides a prediction model to help identify patients who may need high-cost imaging tests, such as MRI or CT, and potentially surgical treatment due to worsening knee pain in the future.

## Materials and methods

### Study design

Our study included participants from the fifth Korea National Health and Nutrition Examination Survey (KNHANES), an annual survey that was conducted between 2010 and 2012. The KNHANES is a cross-sectional survey designed to assess the health and nutritional status of Koreans that has been conducted since 1998. The Korea Disease Control and Prevention Agency oversees the KNHANES by selecting a representative sample of approximately 10,000 noninstitutionalized civilians each year.

**Fig 1:**
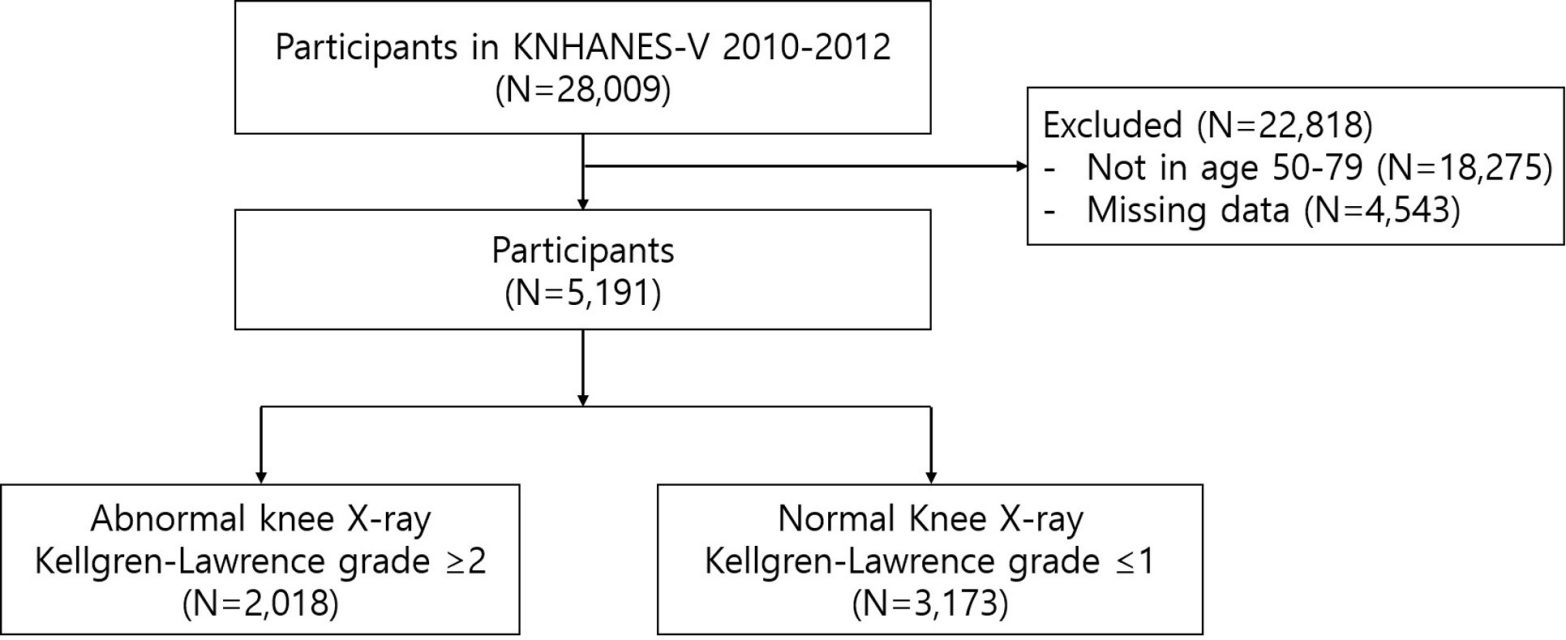
Flow diagram of study participants in the fifth Korea National Health and Nutrition Examination Survey.

Fig 1 illustrates the study design, which began with 28,009 participants from the 2010–2012 KNHANES. During the KNHANES, participants were asked about knee symptoms and a radiological classification of the knee was performed. Participants with missing or inaccurate data, including those who did not undergo knee radiography (N = 4,543), and those aged > 80 or < 50 years (N = 18,275) were excluded from the study. Eventually, we analyzed the data of 5,191 participants aged 50–79 years. The target population for this study ranged from 50 to 79 years old. This decision was based on the fact that the KNHANES survey inquired about knee pain only for individuals aged 50 and older, while those aged 80 and above were grouped into a single category (80 years and older) for reporting purposes. Therefore, we determined that analyzing the age group from 50 to 79 would be optimal and focused our analysis solely on this range. Additionally, it is important to note that the KNHANES is a national representative database, constructed by randomly visiting households, with about 10,000 individuals selected annually from South Korea’s 50 million citizens. Given this random assignment process, the likelihood of both partners from the same household participating in the survey is extremely low, ensuring the independence of data.

This study was based on the KNHANES database and approved by the Institutional Review Board of the Korea Disease Control and Prevention Agency. We obtained exemption for informed consent from the Public Institutional Review Board designated by the Ministry of Health and Welfare (IRB No. 2024-0127-001; Approval No. P01-202401-01-043) All consents were considered and done in the KNHANES.

### Characteristics of the KNHANES database

**Fig 2:**
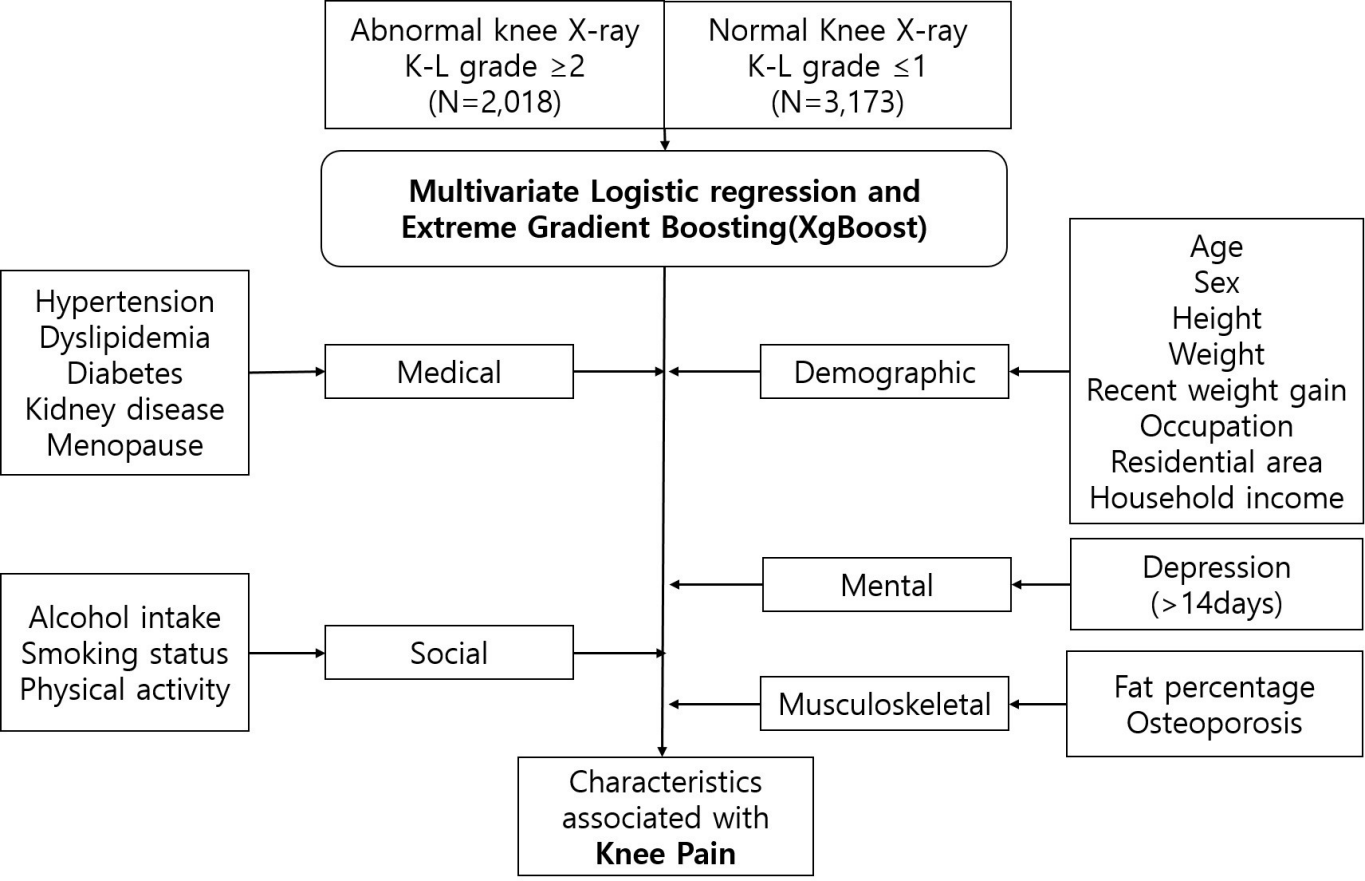
Characteristics of participants and schema of analyses used in this study.

This section describes the key characteristics of the Korean National Health and Nutrition Examination Survey (KNHANES) database, which includes demographic, health, and nutritional information collected from a representative sample of the Korean population. For our study, we used the health interviews, health examination surveys, and dual-energy X-ray absorptiometry (DEXA) for body composition to investigate the characteristics of knee pain [14]. Health interview data, such as epidemiological factors, were obtained through questionnaires; whereas health examination surveys, including height measurements, were conducted by national organizations. Musculoskeletal factors, including fat percentage and osteoporosis measured by DEXA and obtained at the mobile examination center. Fig 2 displays all variables examined in this study.

The demographic factor "recent weight gain" refers to individuals who reported an increase in body weight over the past year by answering "yes" to the question, "Have you gained weight compared to one year ago?" Occupation was categorized into two groups: white-collar jobs, including office and professional roles, and blue-collar jobs, representing manufacturing and labor roles. Residential area was classified into residents living in cities versus those in rural areas within Korea. Household income was divided based on whether individuals were in the top 50% of income earners or not.

Medical and mental factors encompass diagnosed illnesses from health surveys conducted by trained personnel. The KNHANES survey includes current diagnoses of conditions such as hypertension, dyslipidemia, diabetes, and kidney disease. Additionally, depression is considered a risk factor, defined by a persistent depressive mood lasting over two weeks.

The KNHANES datasets provide whole-body DEXA measurements, including bone mineral density, muscle mass, and fat mass. In this study, fat percentage derived from DEXA measurements was used in the analysis as a musculoskeletal factor [14, 15]. In this study, diagnosis of osteoporosis was defined as a binary outcome for individuals who answered "yes" to the question, "Have you ever been diagnosed with osteoporosis by a doctor?" Additionally, we included individuals whose DEXA scan T-scores were measured at -2.5 or lower.

Moreover, social factors were evaluated using questionnaires covering smoking, alcohol consumption, and physical activity, all of which directly impact an individual’s health status. Smoking was quantified in pack-years as a cumulative measure over a person’s lifetime [16]. Similarly, alcohol intake was determined by the weekly average amount of alcohol consumed, expressed in Soju bottles. Soju, a prevalent alcoholic beverage in Korea, typically comes in 360ml bottles with an alcohol content of approximately 16% [17]. Physical activity was calculated based on parameters from KNHANES questionnaires, converted into metabolic equivalents (METs) [18].

Knee radiographs were graded using the Kellgren–Lawrence (K-L) grading system, with bilateral weight-bearing anteroposterior and lateral knee radiographs taken using an SD3000 Synchro Stand (Accele Ray Shinyoung Co., Seoul, Korea). K-L grades were defined as follows: grade 1 is defined as doubtful narrowing of joint space and possible osteophyte formation; grade 2 represents definite osteophytes and possible joint space narrowing; grade 3 shows multiple osteophytes, definite joint space narrowing, and some sclerosis; and grade 4 is characterized by large osteophytes, severe joint space narrowing, marked sclerosis, and possible bony deformity. Two radiologists independently conducted osteoarthritis examinations, with a higher grade accepted in cases of a one-grade difference between them. Discrepancies exceeding one grade were reviewed by a third radiologist, and the grade assigned by the third assessment was utilized. The interrater agreement within one grade of difference between the two radiologists was 92.8%, with a weighted Cohen’s κ coefficient of 0.65 [14, 19]. Knee pain was characterized as the presence of pain in the knee lasting for at least 30 days within the preceding three months. The intensity of pain was assessed using a numeric rating scale (NRS) ranging from 0 to 10 points. In cases where pain levels differed between both knees, the severity of the more intense side was noted. The KNHANES data only assigned grades to the affected joint, without considering both knees. Similar data collection methods and analyses have also been used to assess osteoarthritis in other joints, such as the hip joint. Consistent with prior research, Kellgren–Lawrence grades 2 to 4 were classified as abnormal X-ray findings, whereas grades 0 to 1 were considered indicative of normal X-ray findings [20].

### Statistical analysis

#### Descriptive analysis

Since knee pain-related diseases, such as osteoarthritis and meniscus tears, are significantly associated with sex [6], the baseline characteristics of the participants were analyzed based on their categorization into male and female groups. Comparable analyses were performed by categorizing participants into age groups: 50–59, 60–69, and 70–79. Variables such as continuous measures, including weight, were presented as means with standard deviations and were compared using Student’s t-tests. Categorical variables, such as radiographic grades, were expressed as percentages and counts, with comparisons conducted using the chi-squared test. Normality tests, such as the Shapiro-Wilk test and Kolmogorov-Smirnov test (depending on data size), were applied to all variables. In cases where normality assumptions were not satisfied, non-parametric tests, such as the Mann-Whitney U test, were utilized for the respective variables.

#### Multivariate logistic regression

We investigated the discrepancies between radiological knee grade and patient-reported pain. Only data from the more affected knee were used for each participant, specifically the knee with the higher K-L grade. Participants with normal knee X-ray findings or abnormal knee X-rays were analyzed using multivariate logistic regression to identify factors linked to knee pain. The model included the following covariates: age, sex (female), body mass index (BMI), weight gain in the past year, fat percentage, diagnoses of hypertension, dyslipidemia, diabetes, nephrotic disease, and osteoporosis, depressive mood (lasting more than 14 days), menopause status, occupation type (blue-collar worker), household income (top 50%), residential area (rural living), alcohol intake, smoking amount, and physical activity (measured in METs). To minimize multicollinearity among the multiple regression variables and to improve the robustness and validity of the analysis, we opted to use only BMI among the variables of height, weight, and BMI. This decision was based on previous research that has established a significant association between knee pain and BMI [21].

#### Extreme gradient boosting

Machine learning prediction models are extensively used in social science and medicine [22, 23]. To demonstrate knee pain prediction, we utilized XGBoost, a powerful gradient-boosting algorithm that enhances classification and regression models. XGBoost is based on an ensemble of decision trees, using a gradient descent algorithm to minimize errors by iteratively adding trees that correct residuals from previous models. This approach leverages both the strengths of tree-based models and the mathematical principle of gradient descent, offering a compact, error-minimizing model that is well-suited for prediction tasks in medical fields, as it optimizes model performance by reducing prediction error with each iteration [13]. The XGBoost model was optimized in two stages: first, hyperparameters were fine-tuned using grid search, and then the model was trained and validated through 100 iterations to estimate performance confidence intervals.

Hyperparameters optimized via grid search included tree depth (1, 2, 3, 5, 10, 15), learning rate (0.1, 0.25, 0.5, 0.75), gamma (0.1, 0.5, 1, 2, 3), subsample ratio of training instances (0.5, 0.75, 1), minimum instance weight (1, 2, 3), and column subsample ratio (0.75, 1). Approximately 2,160 hyperparameter combinations were evaluated, with root mean square error (RMSE) used to compare model performance.

To prevent overfitting, five-fold cross-validation was employed. The dataset was divided into five groups, with each group serving as a test set in turn while the remaining four groups were used as training sets. RMSE was averaged over the five iterations to identify the optimal hyperparameters.

The model with the smallest RMSE was selected as having the optimal hyperparameters. For normal knee X-ray groups, the best parameters were a learning rate of 0.25, tree depth of 1, gamma of 3, column subsample ratio of 1, minimum instance weight of 3, and a training instance subsample ratio of 0.75. For abnormal knee X-ray groups, the optimal settings were a learning rate of 0.1, tree depth of 1, gamma of 1, column subsample ratio of 1, minimum instance weight of 3, and a training instance subsample ratio of 0.5.

The optimized XGBoost model was trained with 70% of the data for predicting knee pain and tested on the remaining 30%. The cutoff value was determined using Youden’s index [24]. Model performance was assessed using the area under the receiver operating characteristic curve (AUC). An AUC of 0.5 indicates no predictive power, while higher values reflect better accuracy [25]. Additional metrics, including accuracy, precision, recall, and F1 score, were also evaluated.

An advantage of XGBoost is its feature importance function, which ranks variables based on their significance in the prediction process. Feature importance was assessed using the "gain" metric, which measures each variable’s contribution to the model’s predictions [26]. Higher gain values indicate greater importance. To account for variability due to random initialization in XGBoost, we performed 100 iterations to provide robust estimates with 95% confidence intervals [27]. Early stopping was not implemented in this article.

All analyses were performed using R version 4.2.2 on the Windows 10 operating system and various R libraries, including ’dplyr,’ ‘moonBook,’ ‘xgboost,’ and related packages. The level of significance was set at p < 0.05.

## Results

We conducted a comprehensive analysis of the participants’ characteristics, an overview of which is presented in Table 1. The findings indicate that female participants reported a higher incidence of knee pain (36.8% vs. 15.8%), elevated knee pain NRS scores (2.5±3.4 vs. 1.0±2.3), and more abnormal K-L grades (45.7% vs. 29.9%). The data revealed significant differences between the sexes in terms of medical conditions, including diagnosis of depression (10.2% in males vs. 20.6% in females), dyslipidemia (12.8% in males vs. 19.6% in females), and diabetes (16.4% in males vs. 13.3% in females). Further distinctions were observed in demographic factors, such as height (166.8±5.8 cm in males vs. 153.7±5.7 cm in females), weight (66.0±9.6 kg in males vs. 57.5±8.6 kg in females), BMI (23.7±2.9 in males vs. 24.3±3.2 in females), fat percentage (22.6±5.1% in males vs. 34.5±5.4% in females), and recent weight gain in 1 year (6.7% in males vs. 12.5% in females). Social factors such as alcohol intake (number of soju bottles consumed per week: 1.4±2.0 in males vs. 0.1±0.5 in females), smoking status (pack-years: 23.8±21.8 in males vs. 0.9±4.8 in females), and physical activity (METs: 306.2±507.8 in males vs. 251.9±487.2 in females) showed significant differences between sexes. However, no significant differences were noted in terms of age, diagnosis of hypertension or nephrotic disease, and living area between the sexes.

**Table 1:** Descriptive analysis of the study participants by sex.

	Male (N = 2,252)	Female (N = 2,939)	p-value
Age	63.1±8.1	62.7±8.4	0.123
Height (cm)	166.8±5.8	153.7±5.7	<0.001
Weight (kg)	66.0±9.6	57.5±8.6	<0.001
BMI	23.7±2.9	24.3±3.2	<0.001
Recent weight gain in 1 year	6.7%	12.5%	<0.001
Fat percentage	22.6±5.1	34.5±5.4	<0.001
Diagnosis of hypertension	37.6%	40.1%	0.075
Diagnosis of dyslipidemia	12.8%	19.6%	<0.001
Diagnosis of diabetes	16.4%	13.3%	<0.001
Diagnosis of nephrotic disease	0.4%	0.5%	0.757
Diagnosis of osteoporosis	2.0%	20.5%	<0.001
Depressive mood (>14 days)	10.2%	20.6%	<0.001
Menopause	0	92.1%	<0.001
Occupation type (blue-collar)	52.8%	33.6%	<0.001
Household income (top 50%)	46.0%	39.3%	<0.001
Residential area (living in rurality)	30.5%	30.0%	0.703
Alcohol intake	1.4±2.0	0.1±0.5	<0.001
Smoking amount	23.8±21.8	0.9±4.8	<0.001
Physical activity (METs)	306.2±507.8	251.9±487.2	<0.001
Knee pain	15.8%	36.8%	<0.001
Knee pain NRS score	1.0±2.3	2.5±3.4	<0.001
Abnormal knee X-ray	29.9%	45.7%	<0.001

All variables are presented as mean±standard error or frequency (%). BMI, body mass index; Depressive mood, feeling depressed for more than 2 weeks; Alcohol intake, number of soju bottles consumed per week; Smoking amount, average number of cigarette packs smoked per day was based on the number of years of smoking; Physical activity, metabolic equivalents based on questionnaires; and Abnormal knee X-ray, radiological grade 2 or higher knee OA based on the Kellgren–Lawrence grading system.

This study also examined the age distribution of participants, dividing them into three groups: 50–59, 60–69, and 70–79 (see Table 2). As anticipated, older individuals demonstrated a higher prevalence of medical conditions, including hypertension, diabetes, and osteoporosis. Older age was associated with various factors, including decreased height and lower weight. It was also linked to reduced involvement in blue-collar occupations and lower income. Regarding social factors, physical activity and alcohol consumption decreased with age, while smoking rates slightly increased. Additionally, knee pain sensation and abnormal knee X-ray were found to be correlated with aging.

**Table 2:** Descriptive analysis of the study participants by age groups.

	Aged 50–59 (N = 2,022)	Aged 60–69 (N = 1,849)	Aged 70–79 (N = 1,320)	p-value
Age	54.3±2.8	64.5±3.0	73.8±2.7	<0.001
Sex (female)	58.9%	54.2%	56.5%	<0.05
Height (cm)	161.1±8.4	159.5±8.3	156.6±9.0	<0.001
Weight (kg)	62.9±9.9	61.6±9.5	58.1±9.9	<0.001
BMI	24.2±3.0	24.2±3.0	23.6±3.3	<0.001
Recent weight gain	14.6%	8.1%	5.6%	<0.001
Fat percentage	66.2±7.3	66.6±7.5	66.5±7.6	0.201
Diagnosis of hypertension	26.5%	43.9%	51.4%	<0.001
Diagnosis of dyslipidemia	16.0%	20.0%	13.0%	<0.001
Diagnosis of diabetes	9.2%	16.3%	20.5%	<0.001
Diagnosis of nephrotic disease	0.3%	0.5%	0.7%	0.387
Diagnosis of osteoporosis	6.7%	14.1%	19.1%	<0.001
Depressive mood (>14 days)	17.3%	15.1%	15.6%	0.161
Menopause	48.7%	53.3%	55.8%	<0.001
Occupation type (blue-collar)	47.4%	44.7%	29.7%	<0.001
Household income (top 50%)	63.0%	36.0%	19.2%	<0.001
Residential area (living in rurality)	23.6%	31.4%	38.6%	0.703
Alcohol intake	0.9±1.7	0.7±1.4	0.5±1.2	<0.001
Smoking amount	9.3±16.4	11.5±19.7	12.1±20.0	<0.001
Physical activity (METs)	334.0±518.4	275.7±511.9	185.5±423.5	<0.001
Knee pain	18.3%	29.6%	39.4%	<0.001
Knee pain NRS score	1.2±2.4	1.9±3.1	2.8±3.6	<0.001
Abnormal knee X-ray	21.8%	43.8%	58.2%	<0.001

All variables are presented as mean±standard error or frequency (%). BMI, body mass index; Depressive mood, feeling depressed for more than 2 weeks; Alcohol intake, number of soju bottles consumed per week; Smoking amount, average number of cigarette packs smoked per day was based on the number of years of smoking; Physical activity, metabolic equivalents based on questionnaires; and Abnormal knee X-ray, radiological grade 2 or higher knee OA based on the Kellgren–Lawrence grading system.

In this study, multivariate logistic regression was conducted to identify significant factors associated with knee pain. Table 3 summarizes these factors, categorized into groups based on normal and abnormal knee X-rays. Several factors, including age, sex, BMI, osteoporosis diagnosis, depressive mood, and residential area (rurality), showed significant associations with knee pain in both groups. Notably, in the group with normal knee X-rays, higher fat percentage is significantly associated with a lower likelihood of knee pain, with an odds ratio (OR) of 0.973 (95% CI: 0.950–0.995).

**Table 3:** Factors associated with knee pain in participants showing two types of knee radiographs.

	Normal knee X-ray (N = 3,173)		Abnormal knee X-ray (N = 2,018)	
	Odds ratio	p-value	Odds ratio	p-value
Age	1.039 (1.024–1.054)	<0.001	1.047 (1.031–1.062)	<0.001
Sex (female)	3.302 (1.926–5.599)	<0.001	3.824 (1.973–7.389)	<0.001
BMI	1.074 (1.030–1.120)	<0.001	1.098 (1.054–1.144)	<0.001
Recent weight gain in 1 year	1.215 (0.897–1.630)	0.200	1.037 (0.741–1.447)	0.828
Fat percentage	0.973 (0.950–0.995)	0.021	0.984 (0.960–1.007)	0.190
Diagnosis of hypertension	0.970 (0.781–1.201)	0.783	1.032 (0.843–1.262)	0.757
Diagnosis of dyslipidemia	1.041 (0.789–1.350)	0.759	0.916 (0.706–1.185)	0.507
Diagnosis of diabetes	0.842 (0.619–1.133)	0.266	1.233 (0.955–1.590)	0.106
Diagnosis of nephrotic disease	1.383 (0.343–4.667)	0.618	0.702 (0.181–2.328)	0.576
Diagnosis of osteoporosis	1.580 (1.196–2.078)	0.001	1.829 (1.404–2.389)	<0.001
Depressive mood (>14 days)	1.746 (1.382–2.197)	<0.001	1.643 (1.270–2.128)	<0.001
Menopause	0.989 (0.659–1.516)	0.958	0.841 (0.474–1.500)	0.554
Occupation type (blue-collar)	1.180 (0.659–1.516)	0.122	1.190 (0.953–1.487)	0.124
Household income (top 50%)	0.828 (0.672–1.018)	0.074	0.873 (0.703–1.083)	0.218
Residential area (living in rurality)	1.252 (1.004–1.557)	0.044	1.306 (1.056–1.617)	0.013
Alcohol intake	0.983 (0.906–1.063)	0.691	1.049 (0.965–1.137)	0.250
Smoking amount	0.999 (0.992–1.006)	0.986	1.000 (0.993–1.008)	0.836
Physical activity (METs)	1.000 (0.999–1.000)	0.441	0.999 (0.999–1.000)	0.659
Intercept	0.003		0.001	

BMI, body mass index; Abnormal knee X-ray, radiological grade 2 or higher knee OA based on the Kellgren–Lawrence grading system; Depressive mood, feeling depressed for more than 2 weeks; Alcohol intake, number of soju bottles consumed per week; Smoking amount, average number of cigarette packs smoked per day was based on the number of years of smoking; and Physical activity, metabolic equivalents based on questionnaires.

Through the feature importance results of the XGBoost model used to predict knee pain, we identified the key characteristics contributing to the prediction. BMI emerged as the most significant predictor of knee pain, with a feature importance score of 0.203±0.003. Following BMI, the next most important predictors were age, sex, depressive mood, fat percentage, physical activity, and smoking amount. The XGBoost model demonstrated an AUC of 0.6741 ± 0.0007, an accuracy of 0.608 ± 0.008, a precision of 0.262 ± 0.003, a recall of 0.679 ± 0.013, and an F1 score of 0.376 ± 0.001. Table 4 highlights these top seven variables for predicting knee pain in participants with normal knee X-rays.

**Table 4:** Feature importance of the XGBoost model for knee pain with normal knee radiographs.

Characteristics	Feature Importance
BMI	0.203±0.003
Age	0.181±0.002
Sex (female)	0.156±0.004
Depressive mood (>14 days)	0.084±0.001
Fat percentage	0.080±0.003
Physical activity (METs)	0.056±0.002
Smoking amount	0.055±0.002

Normal knee X-ray, radiological grade 0 or 1 knee OA based on the Kellgren–Lawrence grading system; BMI, body mass index; Physical activity, metabolic equivalents based on questionnaires; and Smoking amount, average number of cigarette packs smoked per day was based on the number of years of smoking (pack-years).

Using the XGBoost model to predict knee pain in participants with abnormal knee X-rays, we assessed the importance of various characteristics. Age emerged as the most significant predictor, with a feature importance score of 0.187±0.002. This was followed by BMI, sex, diagnosis of osteoporosis, menopause, fat percentage, and physical activity. The XGBoost model demonstrated an AUC of 0.6591 ± 0.0006, an accuracy of 0.620 ± 0.002, a precision of 0.545 ± 0.003, a recall of 0.685 ± 0.012, and an F1 score of 0.605 ± 0.003. Table 5 highlights the top seven most important variables for predicting knee pain in participants with abnormal knee X-rays.

**Table 5:** Feature importance of the XGBoost model for knee pain with abnormal knee radiographs.

Characteristics	Feature Importance
Age	0.187±0.002
BMI	0.151±0.003
Sex (female)	0.125±0.005
Diagnosis of osteoporosis	0.102±0.002
Menopause	0.084±0.006
Fat percentage	0.079±0.004
Physical activity (METs)	0.060±0.002

Abnormal knee X-ray, radiological grade 2 or higher knee OA based on the Kellgren–Lawrence grading system; BMI, body mass index; Physical activity, metabolic equivalents based on questionnaires.

## Discussion

In clinical practice, identifying patients who may experience pain despite normal radiographic results is challenging. Since decisions regarding knee treatments, such as medication or surgery, heavily rely on the patient’s reported pain, accurately predicting knee pain is crucial for effective orthopedic decision-making [28]. This is also important for patients with abnormal knee X-rays, who are often considered for surgical treatment or further evaluation [29]. Accurately predicting knee pain based on various personal factors, such as osteoporosis or depression, can assist clinicians in determining whether to recommend advanced imaging tests, such as MRI, or proceed with surgery. Eventually, predicting knee pain plays an important role in national health insurance expenditure and public health [30].

This study analyzed multiple demographic and medical factors statistically to determine their relationship with knee pain. For both patients with normal and abnormal knee X-rays, common significant variables associated with knee pain included age, female sex, high BMI, diagnosis of osteoporosis, depressive mood, and living in a rural area. For patients with normal knee X-rays, a multivariate logistic regression model found that a higher fat percentage was associated with lower knee pain. Similarly, the XGBoost model identified age, sex and BMI as the top three important factors in both normal and abnormal knee X-ray cases.

Aging and knee pain are associated due to the deterioration of cartilage and bony structures, which become more susceptible to damage and degeneration over time. The articular cartilage that cushions the ends of bones in the knee joint wears down and loses water content, leading to reduced weight support and less effective load distribution [31]. Additionally, increased vulnerability in ligaments and tendons of the knee joint contributes to greater joint instability [32], which results in reduced joint movement and increased pain.

Female sex was identified as a risk factor for knee pain. Previous studies have suggested that the pathophysiology of knee OA and meniscus injury is a key factor in the differences between the sexes. The incidence of knee OA and soft tissue injuries has been reported to dramatically increase in women around menopause. Some studies indicate that estrogens offer protective effects and that hormonal factors, including inflammation, play a role in the onset of these conditions [33–35]. Given that the participants in this study were all over 50 years old, most of the female participants were menopausal, leading to notable differences in characteristics between the sexes.

The observation that a higher BMI correlates with increased knee pain aligns with findings from previous studies. While it is difficult to establish a causal relationship due to the cross-sectional design of this study, one possible explanation is that knee pain may lead to a higher BMI. For example, individuals with knee pain who have a normal weight might adopt a static lifestyle, which could result in reduced activity levels and subsequent weight gain [21]. On the other hand, a high BMI can also induce knee pain, as the knee joint must accommodate and cushion the body weight during movement [36]. Our research similarly identified a significant association between high BMI and knee pain, consistent with earlier studies.

This study suggested that a reduced fat percentage is associated with knee pain, which appears to contrast with previous research. Earlier studies have indicated that higher adipose tissue levels are linked to increased secretion of inflammatory cytokines, which, in turn, are associated with osteoarthritis and heightened knee pain. However, our research is a cross-sectional study and does not establish a causal relationship between fat percentage and knee pain; rather, it describes the association. One possible explanation for our results is that individuals with lower fat percentages may be more physically active, which could lead to cumulative injuries and, consequently, increased knee pain. In fact, as shown in our S1 Table, a statistically significant inverse correlation was observed between fat percentage and physical activity (p < 0.05). Moreover, a low fat percentage and high knee pain were associated in the group with normal X-rays, and since the X-rays showed normal knee structures, it is expected that their activity was not significantly restricted, making it reasonable to link this to high activity levels. If we had conducted a longitudinal study, we might have observed a temporal relationship between the increase in fat percentage and subsequent increases in adipose tissue cytokines, leading to heightened knee inflammation and, consequently, increased knee pain. However, since this is a cross-sectional study, there may be differences in the interpretation of these results. In addition, these findings could be influenced by various factors, such as the complexity of knee pain etiology, the role of other variables that may interact with fat percentage, or the possibility of residual confounding. Also, the small effect size and statistical significance (p = 0.021 for fat percentage) suggest that while there is a statistically significant association, the practical significance may be limited. These findings warrant further investigation in longitudinal studies to better understand the underlying mechanisms and confirm the practical relevance of these associations in real-world settings.

In the XGBoost analysis of participants with normal X-rays, depressive mood was identified as a notable factor for predicting knee pain. Multiple studies have shown that depression can exacerbate mobility issues and increase psychosomatic pain, thereby contributing to knee pain. Additionally, challenges in carrying out daily activities due to knee pain can lead to the development of depressive symptoms [37, 38].

The XGBoost model in participants with abnormal knee X-rays revealed that higher BMI and the presence of osteoporosis can exacerbate knee pain. The mechanical stress on the knee joint can help explain these results. Low bone mineral density reduces the bone’s ability to withstand mechanical stress, such as that imposed by heavier weight (higher BMI), leading to increased pain [36, 39]. Given that individuals with abnormal X-rays show significant deterioration of knee bony structures, decreased bone density and compromised resistance force due to osteoporosis are likely to be more pronounced. Therefore, in this study, weight and osteoporosis are considered to be more significant predictors of knee pain in individuals with abnormal knee X-rays.

Recent weight gain is known to have a significant association with knee pain [40]. In the multivariate logistic regression analysis, recent weight gain was associated with a higher likelihood of knee pain, consistent with previous research. However, in the XGBoost analysis, recent weight gain was not considered a significant factor. Specifically, it ranked 13th out of all variables in the normal X-ray group (feature importance: 0.01±0.0008) and 15th in the abnormal X-ray group (feature importance: 0.002±0.0006). One possible explanation is that our study is cross-sectional, limiting the ability to establish causal relationships. Additionally, feature importance in XGBoost reflects the relative contribution of variables to knee pain prediction. Therefore, variables such as age and BMI, which were identified as more critical in our study, may have taken precedence. For instance, the likelihood of knee pain increasing due to a 1 kg weight gain in a young individual with a normal BMI might be lower compared to an elderly individual with a higher BMI who experiences knee pain, even without recent weight gain.

This study found that residents of rural areas were more likely to experience knee pain. Previous studies in Greece and Thailand reported more severe knee diseases in rural areas, due to a higher proportion of older residents in rural areas and better medical access in urban areas [41, 42]. Analyzing these studies suggests that, in Korea as well, the higher proportion of elderly individuals and reduced medical access in rural areas likely contribute to the association between knee pain and residing in rural areas [43]. The presence of correlations between variables should be considered when interpreting the results. The correlation matrix for all the variables used in our study is presented in S1 Table. For instance, as shown in the matrix, living in a rural area is significantly associated with both higher age and a diagnosis of osteoporosis (p < 0.05), which were linked to knee pain in this study. Recognizing these relationships between variables will aid in achieving a more accurate interpretation of our findings.

Regarding social factors, our study suggests that alcohol intake and smoking status were not related to knee pain sensation. Smoking and alcohol intake result in cartilage loss, modulation of the immune system, chronic inflammation, and worsening of knee diseases [44, 45]. However, our results suggest no significant association, which might be due to inaccuracies in self-reported data based on recall. Alternatively, it is possible that smoking and alcohol intake may not have a significant relationship with knee pain in the Korean population. Further studies are needed to clarify the relationship between knee pain and smoking and alcohol consumption.

Our study had several limitations. First, as a cross-sectional study, it identifies relationships rather than causations, which makes it challenging to establish definitive cause-and-effect links. Second, while knee lesions like osteoarthritis progress along a continuous spectrum, the discrete K-L grades may affect the accuracy of image classifiers, as intermediate cases might not be well-represented. Furthermore, our analysis was limited to individuals aged between 50 and 79 based on KNHANES data, which grouped those aged 80 and older into a single category. Including a broader age range might have provided more nuanced insights. Additionally, the survey did not address bone-forming nutrients like Vitamin D or trauma history, which could have offered deeper insights into knee pain and X-ray results. Self-reported data on smoking and physical activity may introduce biases, including recall bias. In fact, these limitations extend beyond smoking and physical activity; our study also relied on self-reported data for past medical history, including diabetes and hypertension, as well as depression and knee pain itself. This reliance on self-reported data inevitably suggests limitations in our study. Moreover, the lack of questions about previous knee conditions, such as septic knee or total knee arthroplasty, restricted our ability to provide comprehensive information on knee pain and X-ray findings.

In our study, the AUC and accuracy of the XGBoost model were relatively low. One such factor is the cross-sectional nature of the study. Given the cross-sectional design, causal relationships cannot be inferred, which makes it difficult for the model to capture the complex temporal dynamics that could be important for predicting knee pain based on the XGBoost model. Another factor is feature selection and data quality. While we identified key factors such as BMI, age, and physical activity, there may be other variables influencing knee pain that were not included in the KNHANES survey. For instance, factors like a history of knee surgery or steroid injections, which could have a strong association with knee pain, were not included. Additionally, the alignment of the lower extremities is an important factor related to knee pain. When malalignment occurs, such as in genu varus or genu valgus, where the knee joint deviates laterally or medially from the mechanical axis, weight distribution becomes concentrated in specific areas of the knee joint [46]. The presence of such unmeasured variables, combined with potential data noise inherent in the KNHANES dataset, may have reduced the model’s predictive power.

Future studies should aim to include a wider age range, gather data on nutritional intake and trauma history, and obtain detailed information on participants’ knee history. Additionally, it would be beneficial to focus on the performance metrics that are most relevant for each model. For example, for the XGBoost model in the normal knee X-ray group, the priority should be on predicting knee pain, making it crucial to develop a model that minimizes false negatives by emphasizing high recall. Moreover, employing precise and objective measures, such as wearable devices, will enhance the understanding of knee pain and better establish the relationship between physical activity and knee pain.

## Conclusions

For normal X-rays classified as K-L grade 0 or 1 by radiologists, multivariate logistic regression found that knee pain was associated with aging, female sex, higher BMI, lower fat percentage, osteoporosis, depression, and rural living. The XGBoost model identified BMI, age, and sex as key predictors. For abnormal X-rays classified as K-L grade 2 or higher by radiologists, multivariate logistic regression found that knee pain was associated with aging, female sex, higher BMI, osteoporosis, depression, and rural living, while XGBoost highlighted age, BMI, sex, and osteoporosis as significant predictors. Aging and female sex were related to knee pain, possibly due to cartilage and bone changes, as well as hormonal differences. A lower fat percentage was associated with higher pain levels, which might be due to increased activity.

## Supporting information

S1 Table Correlation matrix of all variables used in this study * indicated p<0.05. (DOCX)
