# Supplementary material for: Factors associated with predicting knee pain using knee X-ray and personal factors: A multivariate logistic regression and XGBoost model analysis from the Nationwide Korean Database (KNHANES)
Source: PLoS One. 2024 Dec 2;19(12):e0314789. doi: 10.1371/journal.pone.0314789 (PMC11611080; doi:10.1371/journal.pone.0314789)
Supplement: S1 Table — (DOCX) [file pone.0314789.s001.docx]

S1 Table. Correlation matrix of all variables used in this study

|  | Sex | Age | HTN | DL | DM | CKD | Town | Incm | Dep | Men | Ht | Wt | BMI | reWt | Occp | DEXA | Pain | Pain_s | XR | BW | PY | METs | Fat |
| --- | --- | --- | --- | --- | --- | --- | --- | --- | --- | --- | --- | --- | --- | --- | --- | --- | --- | --- | --- | --- | --- | --- | --- |
| Sex | 1.00 | -0.02 | 0.02* | 0.09* | -0.04* | 0.007 | -0.05 | -0.06* | 0.14* | 0.91* | -0.74* | -0.42* | 0.10* | 0.09* | -0.19* | 0.27* | 0.23* | 0.23* | 0.16* | -0.42* | -0.61* | -0.05* | 0.74* |
| Age | -0.02 | 1.00 | 0.22* | -0.01 | 0.13* | 0.02 | 0.13* | -0.37* | -0.01 | 0.07* | -0.21* | -0.19* | -0.07* | -0.13* | -0.13* | 0.16* | 0.20* | 0.21* | 0.31* | -0.10* | 0.05* | -0.12* | -0.009 |
| HTN | 0.02* | 0.22* | 1.00 | 0.21* | 0.19* | 0.03* | -0.01 | -0.08* | 0.01 | 0.05* | -0.07* | 0.12* | 0.22* | 0.002 | -0.08* | 0.05* | 0.08* | 0.09* | 0.14* | -0.02 | -0.002 | -0.03* | 0.15* |
| DL | 0.09* | -0.01 | 0.21* | 1.00 | 0.17* | 0.04* | -0.09* | 0.03* | 0.04* | 0.10* | -0.04* | 0.08* | 0.14* | 0.02 | -0.11* | 0.06* | 0.02 | 0.02 | 0.02 | -0.05* | -0.05* | -0.01 | 0.13* |
| DM | -0.04* | 0.13* | 0.19* | 0.17* | 1.00 | 0.03* | -0.03* | -0.06* | 0.01 | -0.02 | 0.006 | 0.07* | 0.09* | -0.05* | -0.06* | 0.01 | 0.03* | 0.04* | 0.07* | -0.02 | 0.06* | -0.03* | -0.004 |
| CKD | 0.007 | 0.02 | 0.03* | 0.04* | 0.03* | 1.00 | -0.02* | -0.01 | 0.02 | 0.01 | -0.003 | -0.002 | 0.00 | 0.01 | -0.03* | 0.01 | 0.004 | -0.002 | 0.01 | -0.01* | -0.006 | -0.02 | 0.01 |
| Town | -0.005 | 0.13* | -0.01 | -0.09* | -0.03* | -0.02* | 1.00 | -0.13* | 0.0001 | 0.01 | -0.08* | -0.08* | 0.00 | -0.05* | 0.29* | 0.02* | 0.09* | 0.12* | 0.08* | 0.04* | 0.04* | 0.03* | -0.07* |
| Incm | -0.06* | -0.37* | -0.08* | 0.03* | -0.06* | -0.01 | -0.13* | 1.00 | -0.08* | -0.10* | 0.19* | 0.15* | 0.00 | 0.02* | 0.01 | -0.08* | -0.13* | -0.16* | -0.14* | 0.06* | -0.01 | 0.07* | -0.02 |
| Dep | 0.14* | -0.01 | 0.01 | 0.04* | 0.01 | 0.02 | 0.0001 | -0.08* | 1.00 | 0.12* | -0.12* | -0.09* | -0.01 | 0.04* | -0.04* | 0.06* | 0.11* | 0.13* | -0.001 | -0.03* | -0.05* | -0.002 | 0.08* |
| Men | 0.91* | 0.07* | 0.05* | 0.10* | -0.02 | 0.01 | 0.01 | -0.10* | 0.12* | 1.00 | -0.70* | -0.40* | 0.08* | 0.06* | -0.19* | 0.29* | 0.23* | 0.23* | 0.18* | -0.39* | -0.55* | -0.06* | 0.68* |
| Ht | -0.74* | -0.21* | -0.07* | -0.04* | 0.006 | -0.003 | -0.08* | 0.19* | -0.12* | -0.70* | 1.00 | 0.61* | -0.07* | -0.02 | 0.11* | -0.26* | -0.22* | -0.24* | -0.20* | 0.33* | 0.45* | 0.06* | -0.55* |
| Wt | -0.42* | -0.19* | 0.12* | 0.08* | 0.07* | -0.002 | -0.08* | 0.15* | -0.09* | -0.40* | 0.61* | 1.00 | 0.74* | 0.12* | 0.04* | -0.18* | -0.06* | -0.08* | 0.01 | 0.20* | 0.23* | 0.06* | 0.007 |
| BMI | 0.10* | -0.07* | 0.22* | 0.14* | 0.09* | 0.00 | 0.00 | 0.00 | -0.01 | 0.08* | -0.07* | 0.74* | 1.00 | 0.18* | -0.04 | -0.01 | 0.12* | 0.11* | 0.23* | -0.02 | -0.09* | 0.02 | 0.05 |
| reWt | 0.09* | -0.13* | 0.002 | 0.02 | -0.05* | 0.01 | -0.05* | 0.02* | 0.04* | 0.06* | -0.02 | 0.12* | 0.18* | 1.00 | -0.06* | -0.01 | 0.01 | 0.006 | -0.02 | -0.04* | -0.05* | 0.02 | 0.16* |
| Occp | -0.19* | -0.13* | -0.08* | -0.11* | -0.06* | -0.03 | 0.29* | 0.01 | -0.04* | -0.19* | 0.11* | 0.04* | -0.04 | -0.06* | 1.00 | -0.07* | -0.01 | -0.001 | -0.02* | 0.14* | 0.11* | 0.15* | -0.24* |
| DEXA | 0.27* | 0.16* | 0.05* | 0.06* | 0.01 | 0.01 | 0.02* | -0.08* | 0.06* | 0.29* | -0.26* | -0.18* | -0.01 | -0.01 | -0.07* | 1.00 | 0.17* | 0.18* | 0.08* | -0.13* | -0.16* | -0.03* | 0.20* |
| Pain | 0.23* | 0.20* | 0.08* | 0.02 | 0.03 | 0.004 | 0.09* | -0.13* | 0.11* | 0.23* | -0.22* | -0.06* | 0.12* | 0.01 | -0.01 | 0.17* | 1.00* | 0.81* | 0.26* | -0.10* | -0.13* | -0.02 | 0.19* |
| Pain_s | 0.23* | 0.21* | 0.09* | 0.02 | 0.04* | -0.002 | 0.12* | -0.16* | 0.13* | 0.23* | -0.24* | -0.08* | 0.11* | 0.006 | -0.001 | 0.18* | 0.81 | 1.00 | 0.26* | -0.10* | -0.13* | -0.01 | 0.17* |
| XR | 0.16* | 0.31* | 0.14* | 0.02 | 0.07* | 0.01 | 0.08* | -0.14* | -0.001 | 0.18* | -0.20* | 0.01 | 0.23* | -0.02 | -0.02* | 0.08* | 0.26* | 0.26* | 1.00 | -0.06* | -0.10* | -0.01 | 0.18* |
| BW | -0.42* | -0.10* | -0.02 | -0.05* | -0.02 | -0.01 | 0.04* | 0.06* | -0.03* | -0.39* | 0.33* | 0.20* | -0.02 | -0.04* | 0.14* | -0.13* | -0.10* | -0.10* | -0.06* | 1.00 | 0.35* | 0.03* | -0.33* |
| PY | -0.61* | 0.05* | -0.002 | -0.05* | 0.06* | -0.006 | 0.04* | -0.01 | -0.05* | -0.55* | 0.45* | 0.23* | -0.09* | -0.05* | 0.11* | -0.16* | -0.13* | -0.13* | -0.10* | 0.35* | 1.00 | 0.02* | -0.47* |
| METs | -0.05* | -0.12* | -0.03* | -0.01 | -0.03* | -0.02 | 0.03* | 0.07* | -0.002 | -0.06* | 0.06* | 0.06* | 0.02 | 0.02 | 0.15* | -0.03* | -0.02* | -0.01 | -0.01 | 0.03* | 0.02* | 1.00 | -0.06* |
| Fat | 0.74* | -0.009 | 0.15* | 0.13* | -0.004 | 0.01 | -0.07* | -0.02 | 0.08* | 0.68* | -0.55* | 0.007 | 0.16* | 0.16* | -0.24* | 0.20* | 0.19* | 0.17* | 0.18* | -0.33* | -0.47* | -0.06* | 1.00 |

* indicated p<0.05. HTN, hypertension; DL, dyslipidemia; DM, diabetes mellitus; CKD, kidney disease; Town, living in rural area; Incm, household income (top 50%); Dep, depressive mood over 14 days; Ht, height; Wt, weight; BMI, body mass index; reWt, weight gain in recent 1 year; Occp, occupation (blue collar); DEXA, osteoporosis; Pain, feeling knee pain; Pain_s, subjective pain scoring from 0 to 10; XR, K-L grade of knee X ray; BW, alcohol drinking amount; PY, smoking amount; METs, physical activity; Fat, body fat percentage.
